# ISSLS Prize in Bioengineering Science 2023: Age- and sex-related differences in lumbar intervertebral disc degeneration between patients with chronic low back pain and asymptomatic controls

**DOI:** 10.1007/s00586-023-07542-6

**Published:** 2023-02-18

**Authors:** Noah B. Bonnheim, Ann A. Lazar, Anika Kumar, Zehra Akkaya, Jiamin Zhou, Xiaojie Guo, Conor O’Neill, Thomas M. Link, Jeffrey C. Lotz, Roland Krug, Aaron J. Fields

**Affiliations:** 1 Department of Orthopaedic Surgery, University of California, San Francisco, USA; 2 Department of Epidemiology and Biostatistics, University of California, San Francisco, CA, USA; 3 Department of Radiology and Biomedical Imaging, University of California, San Francisco, CA, USA

**Keywords:** Disc degeneration, Aging, Chronic low back pain, T1ρ (T1rho) magnetic resonance imaging (MRI), Lumbar spine

## Abstract

**Purpose:**

Clinical management of disc degeneration in patients with chronic low back pain (cLBP) is hampered by the challenge of distinguishing pathologic changes relating to pain from physiologic changes related to aging. The goal of this study was to use imaging biomarkers of disc biochemical composition to distinguish degenerative changes associated with cLBP from normal aging.

**Methods:**

T1ρ MRI data were acquired from 133 prospectively enrolled subjects for this observational study (80 cLBP, 53 controls; mean ± SD age = 43.9 ± 13.4 years; 61 females, 72 males). The mean T1ρ relaxation time in the nucleus pulposus (NP-T1ρ; *n* = 650 discs) was used as a quantitative biomarker of disc biochemical composition. Linear regression was used to assess associations between NP-T1ρ and age, sex, spinal level, and study group, and their interactions.

**Results:**

NP-T1ρ values were lower in cLBP patients than controls (70.8 ± 22.8 vs. 76.4 ± 22.2 ms, *p* = 0.009). Group differences were largest at L5–S1 ($\Delta \text{T}1{\text{ρ}}_{\text{cLBP}-\text{control}}=-11.3\;\text{ms}$, *p* < 0.0001), representing biochemical deterioration typically observed over a 9–12 year period (NP-T1ρ declined by 0.8–1.1 ms per year [95% CI]). Group differences were large in younger patients and diminished with age. Finally, the age-dependence of disc degeneration was stronger in controls than cLBP patients.

**Conclusion:**

Aging effects on the biochemical composition of the L5–S1 disc may involve a relatively uniform set of factors from which many cLBP patients deviate. NP-T1ρ values at L5–S1 may be highly relevant to clinical phenotyping, particularly in younger individuals.

## Introduction

The role of intervertebral disc degeneration in chronic low back pain (cLBP), a leading cause of disability and opioid prescriptions [1, 2], is controversial. Patients with cLBP have a higher prevalence of disc degeneration on magnetic resonance imaging (MRI) compared with asymptomatic individuals [3], and there is a strong implication that disc degeneration causes cLBP in a subset of people [4]. However, the clinical significance of disc degeneration in a given individual with cLBP is often ambiguous, and many patients with disc degeneration are diagnosed with non-specific cLBP without a pathoanatomical basis to guide treatment [5].

The link between disc degeneration and pain is further obscured by the complex contributions of biopsychosocial factors [6] and by the challenge of distinguishing pathologic degenerative changes contributing to pain from physiologic changes related to aging [4]. For example, the high prevalence of disc degeneration in asymptomatic individuals [3] and the inability of conventional MRI to distinguish physiologic from pathologic disc aging hamper understanding of cLBP pathogenesis and limit the clinical utility of conventional MRI for cLBP diagnosis and treatment.

In both clinical practice and research, the Pfirrmann grading system is often used to classify *structural deterioration* of lumbar discs on T2-weighted MRI [7]. Prior to structural deterioration, degenerating discs undergo *biochemical deterioration*, including loss of aggrecan, elastin, and type II collagen, and the accumulation of collagen crosslinks [8]. Those biochemical changes are inconspicuous on T2-weighted MRI. As such, current understanding of the role of disc degeneration in cLBP is biased toward the terminal stages of degeneration, and little is known about phenotypes of early disc degeneration in patients with cLBP that are distinct from normal aging.

The goal of this study was to identify imaging biomarkers of disc degeneration that distinguish pathological changes associated with cLBP from normal aging. To do this, subjects with and without cLBP spanning a wide range of ages were imaged with T1ρ MRI. T1ρ relaxation times are positively correlated with glycosaminoglycan content (GAG) and water content in the human nucleus pulposus (NP) [9]. Thus, quantitative imaging biomarkers derived from T1ρ relaxation time maps can provide continuous and objective estimates of disc biochemical composition. Although previous studies have used T1ρ MRI to investigate disc degeneration and low back pain [10–12], this study is the first to directly compare T1ρ MRI biomarkers between a large clinical cohort of patients with non-specific cLBP and asymptomatic controls over a wide age range. In doing so, we sought to discover differences between normal age-related biochemical changes and those associated with cLBP.

## Methods

### Subjects

Between January 2016 and August 2021, 84 patients with cLBP and 54 asymptomatic controls were prospectively recruited with IRB approval. Written informed consent was obtained from each participant. Patients with cLBP were recruited from the non-operative spine service at our institution and included if they met the criteria for cLBP established by the NIH Pain Consortium Research Task Force [13]: low back pain for at least three months and at least half of the days in the past six months. Asymptomatic subjects were recruited via print advertisements and via a cohort identification tool that queries our institution’s electronic health records. Exclusion criteria were a history of spine surgery, radiculopathy consistent with disc herniation, vertebral fracture, ankylosing spondylitis, rheumatoid arthritis, psoriatic arthritis, or malignancy. Patient-reported measures for disability and pain were collected using the Oswestry Disability Index (ODI) and Visual Analog Scale (VAS), respectively.

### MRI

Participants were imaged with 3.0 T MRI using a phased-array spine coil. Sagittal acquisitions of the lumbar spine included clinical fast spin echo images with T1 and T2 weighting and a combined T1ρ/T2 relaxation time mapping sequence (Supplemental Material 1).

### Image analysis

**Fig. 1:**
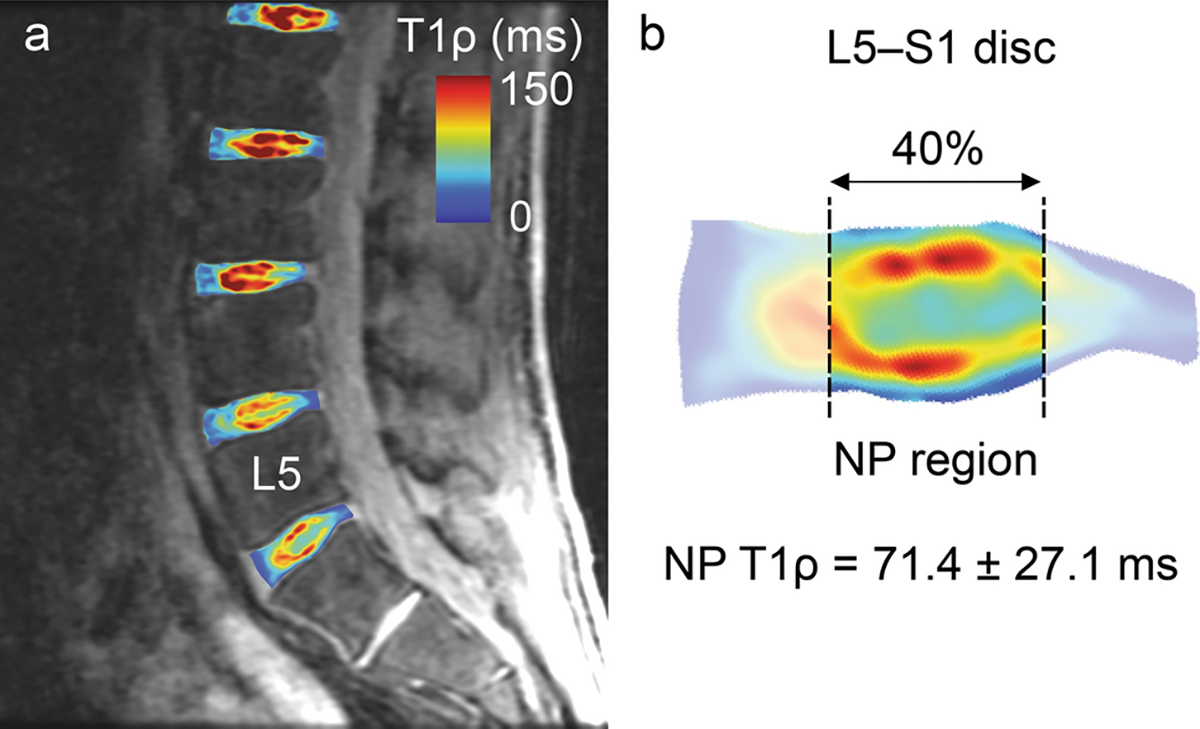
**a** Mid-sagittal T1ρ MRI image showing lumbar disc segmentations and relaxation time maps. **b** The mean ± SD T1ρ relaxation time was computed in the nucleus pulposus (NP) region of each disc

T1ρ relaxation time maps were computed by fitting the signal intensity (SI) decay of each voxel to the mono-exponential decay function: $SI(TLS)=S_{0}e^{TSL/T1\rho }$, where *TLS* is the spin-lock time. After relaxation time mapping, the L1–L2 through L5–S1 discs were segmented from four mid-sagittal T1ρ images using a neural network [14], applying manual corrections as needed (Fig. 1a). Each disc segmentation was rotated into a standard coordinate system using the eigenvalues of the rotational inertia tensor (MATLAB 2020b) to isolate the NP, defined as the central anterior–posterior 40% of the disc (Fig. 1b) [10]. Pfirrmann grading [7] was performed by a radiologist using T2-weighted images.

### Outcomes and statistical methods

The primary outcome of this analysis was the mean T1ρ relaxation time in the NP region of each disc (NP-T1ρ). A mixed effects multivariable linear regression model accounting for multiple observations per subject (*n* = 650 discs from 133 subjects) was used to assess the relationship between NP-T1ρ (outcome) and the following co-variates: age, sex, spinal level, and group (cLBP or control). All nested two- and three-level interactions between co-variates, and the four-level interaction age × sex × level × group, were included in the model. Log transformations and quadratic models were explored but not included based on residual assessment.

A statistically significant (two-sided *p* < 0.05) three-way interaction between age, spinal level, and group (see Results) motivated level-wise linear regression models to test interactions between age and group. These linear regression models included NP-T1ρ (outcome), age, sex, and group. The interaction between age and group was included to assess how the relationship between NP-T1ρ and age differed between groups.

Histograms of all NP voxels were assessed by Pfirrmann grade to examine the relationship between biochemical and structural deterioration.

Using T2 relaxation time mappings, mean NP-T2 values were computed analogously to the methods described for NP-T1ρ (Supplemental Material 2). The statistical tests involving NP-T1ρ were repeated using NP-T2 to assess the generalizability of our results from T1ρ to results from T2 MRI.

Statistical analyses were conducted in JMP Pro (16.0), and two-sided *p* < 0.05 was considered statistically significant. Data are reported as mean ± SD.

## Results

MRI scans were successfully acquired for 133/138 subjects (80/84 cLBP, 53/54 control; Table 1). The L1–L2 disc was outside the field of view for 15/133 subjects. Thus, *n* = 650 discs from 133 participants were included in this study. The cLBP and control groups had similar age and sex distributions (43.9 ± 13.5 vs. 43.9 ± 13.3 years, cLBP vs. control, *p* = 0.98; 44% female and 56% male vs. 49% female and 51% male, *p* = 0.55; Table 1). Subjects had a wide range of disc degeneration: NP-T1ρ ranged from 34.6–148.2 ms and Pfirrmann grade from I–V (Table 1).

**Fig. 2:**
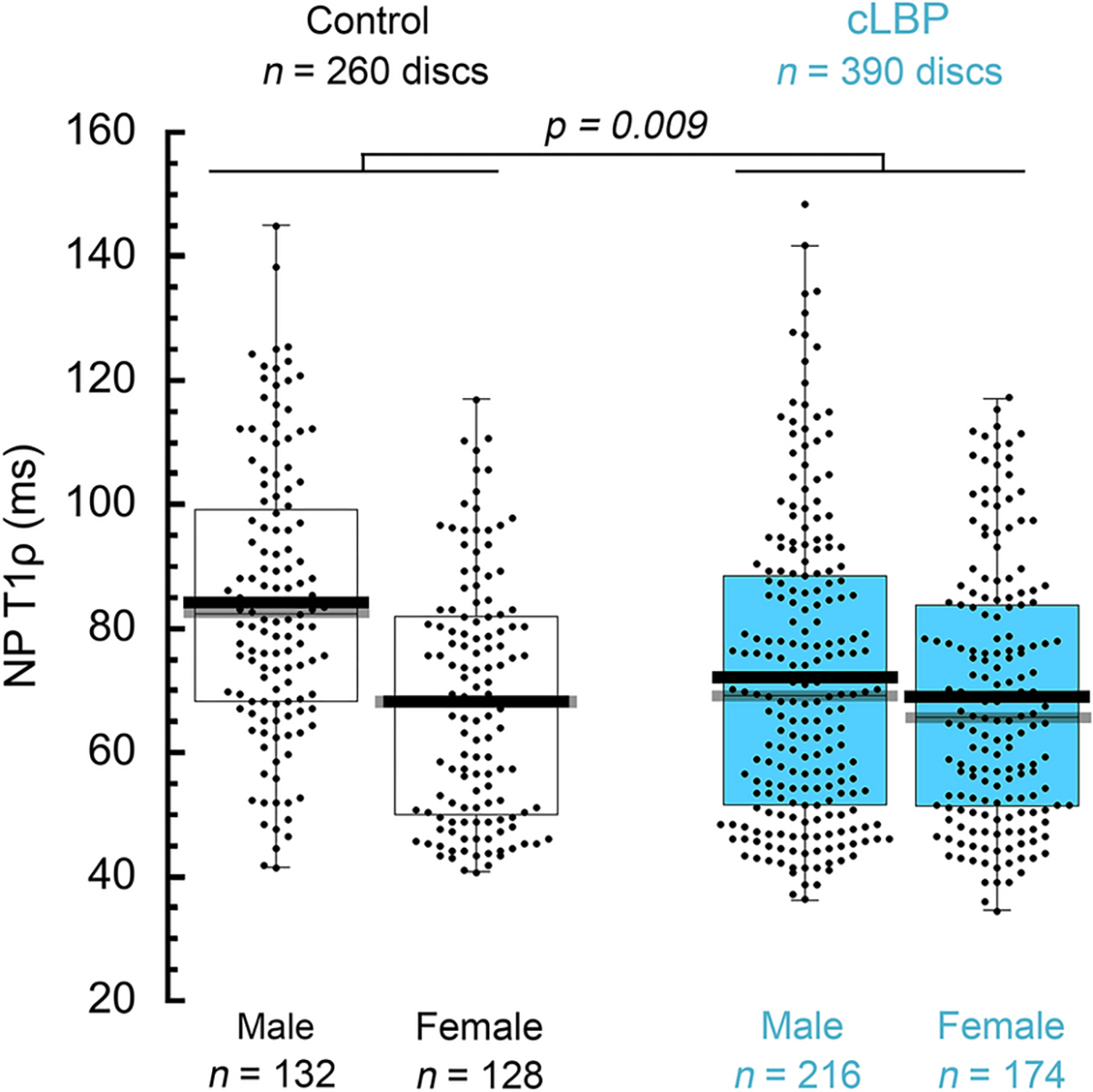
Mean (*black bar*), median (*gray bar*), inter-quartile range (*box*) NP T1ρ distribution (ms) for the control and cLBP groups. In the age- and sex-adjusted model, the cLBP group had lower mean NP-T1ρ values than the control group (*p* = 0.009). Females had lower NP T1ρ values than males (*p* = 0.0001, age-adjusted comparison). The interaction between group and sex was not statistically significant (*p* = 0.15)

**Fig. 3:**
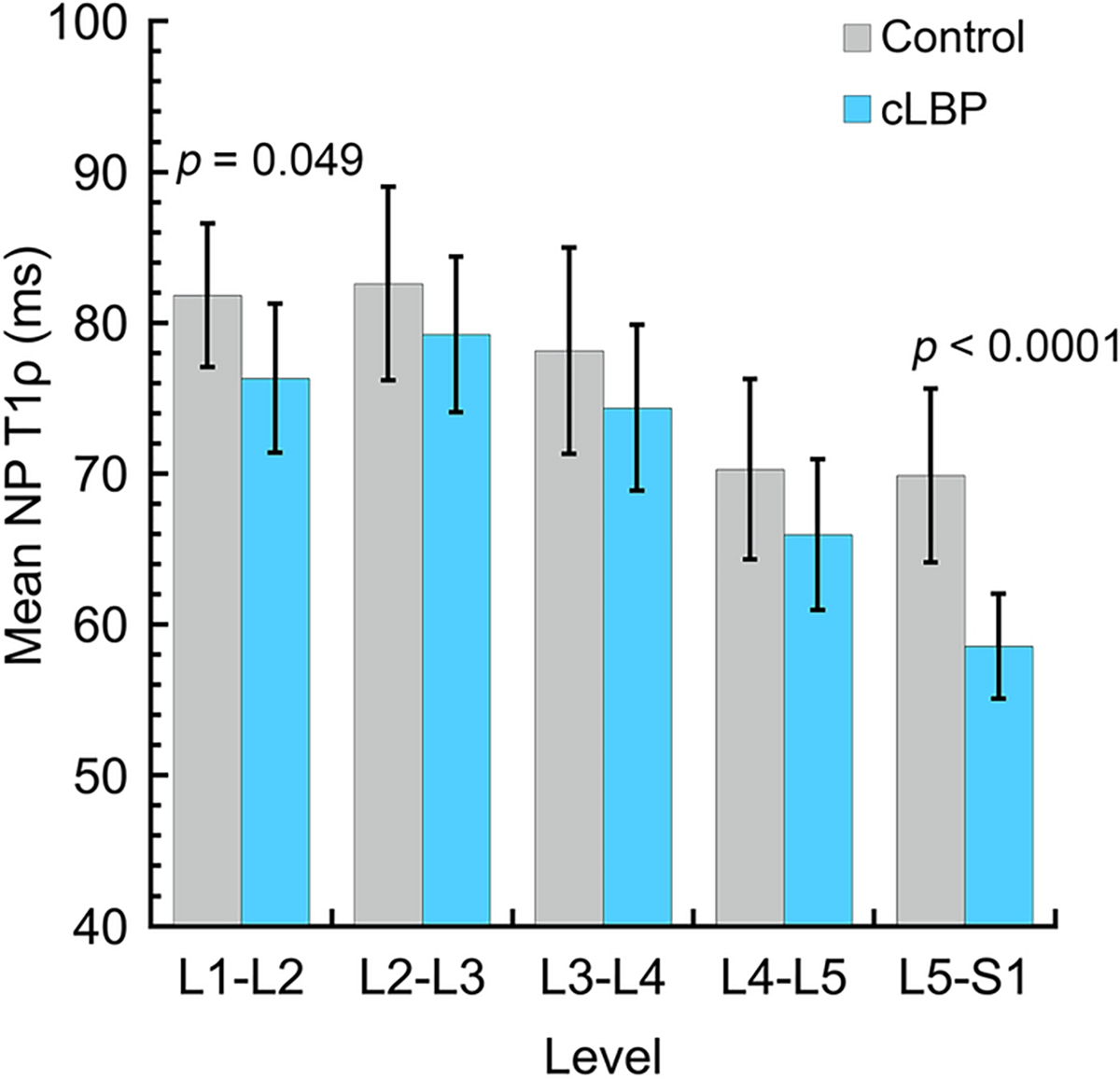
Mean (95% CI) NP-T1ρ by level. *n* = 133 discs per level, except L1–L2, for which *n* = 118 discs

Results from mixed effects regression showed that NP-T1ρ values were significantly lower in the cLBP group than in the control group (70.8 ± 22.8 vs. 76.4 ± 22.2 ms, cLBP vs. control, *p* = 0.009; Fig. 2) and were also lower in females than in males (68.6 ± 20.1 vs. 76.8 ± 24.1 ms, females vs. males, *p* = 0.0001). Sex differences in NP-T1ρ were larger in the control group than in the cLBP group, but this interaction was not statistically significant (*p* = 0.15). NP-T1ρ values were significantly and negatively associated with subject age, decreasing by an average of 1.0 ms per year (95% CI: −0.8 to −1.1 ms, *p* < 0.0001) across the 650 discs. Mean NP-T1ρ values also differed by spinal level (*p* < 0.0001), and were lower in the lower lumbar spine than in the upper lumbar spine (Fig. 3).

Statistically significant two- and three-way interactions between age × level (*p* < 0.0001), and between age × level × group (*p* = 0.02), indicated that relationship between NP-T1ρ and subject age depended on spinal level and study group.

To probe these level-specific effects, regression analyses were conducted at each lumbar level (*n* = 133 discs per level, except L1–L2 [*n* = 118 discs]). Results indicated that mean NP-T1ρ values were lower in cLBP patients than controls at each level L1–L2 through L4–L5, but the differences were relatively small (range: 3.8–5.6 ms, *p* = 0.049–0.19 depending on level, Fig. 3). Conversely, at L5–S1, mean NP-T1ρ values were lower in the cLBP group by 11.3 ms (58.5 ± 20.1 ms vs. 69.8 ± 24.6 ms, cLBP vs. control, *p* < 0.0001).

**Fig. 4:**
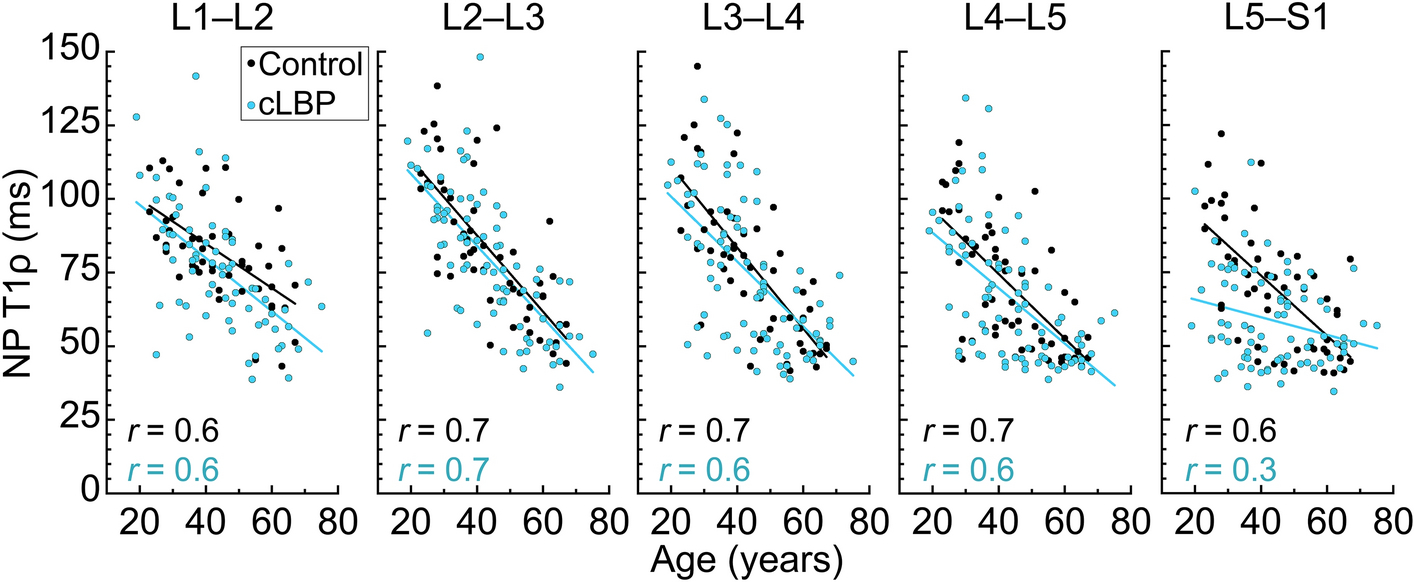
NP-T1ρ as a function of age at each lumbar level (*p* < 0.0001 each except cLBP at L5–S1, for which *p* = 0.02). At L5–S1, there was a statistically significant interaction between age and group (*p* = 0.0008), indicating that the relationship between age and disc degeneration (regression slope) differed between groups. Also at L5–S1, the Pearson’s correlation coefficient for the cLBP group (*r* = 0.3, *p* = 0.02) suggested a weak relationship between age and NP-T1ρ

At levels L1–L2 through L4–L5, the relationships between NP-T1ρ and age were similar in both groups, *i.e.,* similar regression slopes (*p* > 0.33; Fig. 4). However, at L5–S1, there was a statistically significant interaction between age and group (*p* = 0.0008), indicating that the age-dependence of disc degeneration differed between groups. Specifically, the cLBP group had a lower regression slope than the control group (ΔT1ρ = −0.3 ms/year cLBP vs. −1.0 ms/year control).

In the asymptomatic group, NP-T1ρ was strongly correlated with age at all levels (Pearson’s correlation coefficient [*r*] = 0.6–0.7, *p* < 0.0001 each; Fig. 4). The cLBP group also exhibited strong correlations at all levels (*r* = 0.6–0.7, *p* < 0.0001), except for at L5–S1, where the correlation was weak (*r* = 0.3, *p* = 0.02). At L5–S1, group differences in NP-T1ρ were larger in younger subjects and decreased with increasing age.

**Fig. 5:**
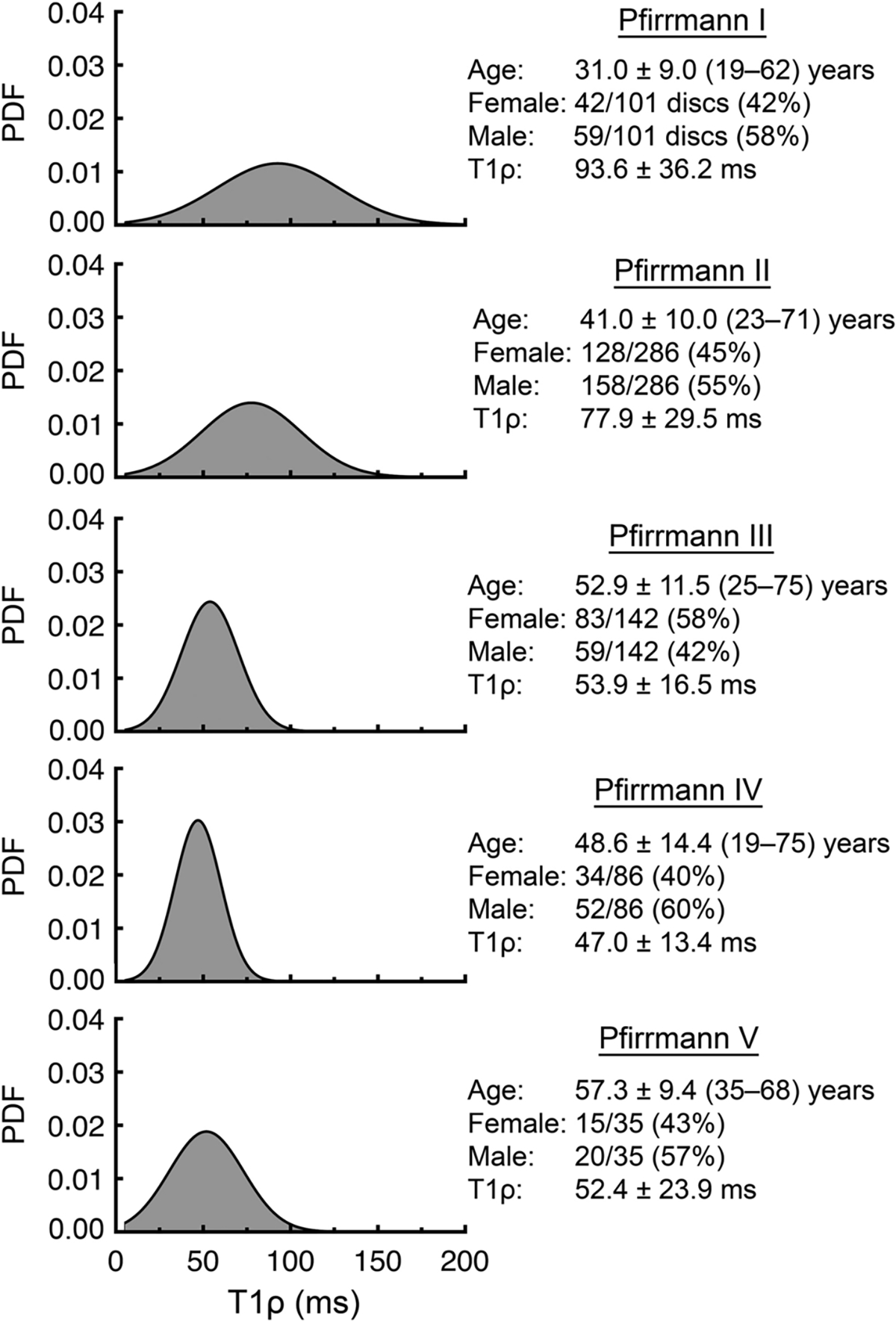
Histogram probability density functions (PDF) for T1ρ relaxation times according to disc Pfirrmann grade for all NP voxels (*n* = 146,387 voxels from 650 discs). Data are mean ± SD (range) or number (percent of total)

NP-T1ρ values were significantly and negatively correlated with Pfirrmann grade (*p* < 0.0001). Assessment of voxel-wise T1ρ relaxation times in the NP (*n* = 146,387 voxels) demonstrated that T1ρ values were more heterogeneous during the early stages of disc degeneration (Pfirrmann grades I and II) compared with later stages of degeneration (Pfirrmann grades III–V; Fig. 5).

Finally, voxel-wise correlations between T1ρ and T2 relaxation times were strong (R^2^ = 0.80, *p* < 0.0001); however, owing to the imperfect nature of the relationship between T1ρ and T2 relaxation times, multivariable regression models predicting NP-T2 differed than models predicting NP-T1ρ (Supplemental Material 2).

## Discussion

In this study, we describe differences in lumbar disc degeneration between a clinical population of patients with non-specific cLBP and asymptomatic controls. Using the mean NP-T1ρ relaxation time as a quantitative biomarker of biochemical deterioration, we found group differences in both the overall extent of disc degeneration and in the role of aging. At L5–S1, we found substantially lower NP-T1ρ values in cLBP patients than controls in age-adjusted models, suggesting that age-adjusted NP-T1ρ values could be used to distinguish pathologic degenerative changes associated with cLBP from normal disc aging, particularly in younger individuals. We also found sex differences indicating that males have higher NP-T1ρ values than females for a given age. Finally, our new data corroborate high levels of biochemical heterogeneity within structurally intact lumbar discs. Our results do not provide evidence that pain in the cLBP group was discogenic, but rather that biochemical disc degeneration was more severe in cLBP patients than controls, and that the difference depended on age and spinal level.

The cLBP group exhibited lower NP-T1ρ values compared with controls in an age- and sex-adjusted model, implying accelerated biochemical disc degeneration in patients with cLBP. These findings are supported by a meta-analysis using imaging findings of structural degeneration [3]. Differences in NP-T1ρ values between study groups were much larger at L5–S1 than at other levels, consistent with higher levels of L5–S1 involvement in discography-concordant pain [15].

Interestingly, the age-dependence of disc degeneration at L5–S1 was stronger in controls than cLBP patients, both in terms of the slope and the amount of variance explained by age. This finding is important because it suggests that aging effects on the disc may involve a relatively uniform set of factors from which many cLBP patients deviate. Factors influencing such deviations are unknown, but could include different etiologies of disc degeneration, e.g., biomechanical/traumatic, genetic, infective/inflammatory, and/or nutritional.

Our results underscore the importance of assessing degenerative phenotypes relative to an individual’s age. To illustrate this, consider an NP-T1ρ value of 60 ms measured at L5–S1 in a 30 year-old individual. That mean T1ρ value would be highly atypical in an asymptomatic 30-year-old based on our study sample (the regression-predicted NP-T1ρ value in asymptomatic people aged 30 years was 84 ms). However, by age 50, an NP-T1ρ value of 60 ms is typical in people with and without cLBP (the regression-predicted NP-T1ρ value in people aged 50-years with and without cLBP was 57 ms and 64 ms, respectively). Thus, the extent of disc degeneration as measured using NP-T1ρ biomarkers may be more relevant to clinical phenotyping when assessed relative to a person’s age; specifically, biochemical deterioration observed at L5–S1 is more important in younger individuals than older ones.

In age-adjusted models, we found higher lumbar NP-T1ρ values in males than females, implying higher amounts of proteoglycan. Higher NP-T1ρ values in males were reported previously [12]. Nonetheless, our present findings are surprising since sex differences in disc health in humans are not commonly reported and because histopathology and MRI studies report contradictory evidence [16, 17]. Males and females exhibit differences in lumbar spine anatomy [18], including differences in lumbar lordosis, vertebral morphology, and muscle mass—those anatomical differences could presumably include and/or impact disc composition.

The mean age-adjusted difference in NP-T1ρ between males and females $\left(\Delta T1\rho_{\text{males-females}}=8.1\;ms\right)$ was equivalent to the degenerative biochemical differences typically observed over a 6–9 year period (NP-T1ρ declined with age by 0.8–1.1 ms per year [95% CI]). This finding is consistent with higher age-adjusted levels of cLBP prevalence and severity in females compared to males [19–21]. Studies in rats demonstrate sex differences in disc degeneration, pain, and healing response, which have been suggested as potential sources for increased pain prevalence in females [22]. To our knowledge, sex differences in proteoglycan content have not been previously reported.

We found substantial heterogeneity in NP-T1ρ values in Pfirrmann grade I discs, consistent with prior findings [12]. Such heterogeneity could reflect differing amounts of biochemical deterioration occurring prior to structural deterioration, or differing peak levels of disc health. Aavikko et al. [23] followed subjects from ages 8 to 34 years and found that lower levels of disc health measured after the pubertal growth spurt in healthy children were associated with low back pain at age 34. Together with our finding that group differences at L5–S1 were large in young patients and diminished with age, the evidence collectively suggests that peak disc health may differ between individuals, possibly from genetic and/or lifestyle factors [4]. This is analogous to hard-tissues: peak bone mass is highly heterogenous and influences the risk of osteoporotic fracture later in life [24]. As such, it is intriguing to consider if development of a T1ρ-based ‘T-score’ could eventually help assess the risk of future cLBP and/or pathologic sequelae of disc degeneration (e.g., Modic changes, herniation, stenosis, etc.). Inter- and intra-individual heterogeneity in peak NP-T1ρ values, and the role of such heterogeneity in future disc degeneration, remains unknown. Nonetheless, our findings point to large variations in disc composition prior to structural damage and relatively small variations during structural deterioration.

Finally, we found that despite strong correlations between T1ρ and T2 relaxation times, the models generated using these MRI techniques did not yield identical statistical conclusions. T1ρ MRI appears advantageous in detecting group differences, likely due to its larger dynamic range and greater sensitivity to GAG compared to T2 MRI [25].

Our study has several limitations. First, the cross-sectional study design prevented assessment of longitudinal changes. Related, while results suggest that disc degeneration at L5–S1 may provide a promising target for distinguishing pathologic degeneration from normal aging, larger studies including adolescents are required to characterize the trajectory and variance in such behavior and to determine diagnostic cut-points. Third, we did not stratify cLBP patients by pain severity or duration and so did not assess the sensitivity of our findings to variations in clinical symptoms. Finally, the statistical models did not include BMI, as BMI data were only available in 39/53 asymptomatic individuals. The cLBP patients had a higher mean BMI than controls (Table 1). However, NP-T1ρ was not associated with BMI in the cLBP group (*p* = 0.19, age-adjusted), suggesting that incorporating BMI data would not alter the overall study conclusions.

In summary, we found several differences in lumbar disc degeneration between patients with non-specific cLBP and asymptomatic controls. Group differences were largest at L5–S1, particularly in younger individuals. Moreover, the age-dependence of biochemical disc degeneration at L5–S1 differed between cLBP patients and controls, suggesting that physiologic disc degeneration follows a relatively uniform course from which those with cLBP tend to deviate. Importantly, the extent of degenerative biochemical changes at L5–S1 converged in cLBP patients and controls with increasing age. We conclude that NP-T1ρ biomarkers at L5–S1, used in multivariate prediction/classification models incorporating sex, may be highly relevant to clinical phenotyping, particularly in younger individuals.

## Supplementary Material

1895942_Sup2

1895942_Sup1

## Figures and Tables

**Table 1:** Subject demographic and clinical data for the 133 participants (*n* = 650 lumbar discs) in this study

Characteristic Sample size	Control *n* = 53 people	Chronic low back pain *n* = 80 people	*p*-value

Age (years)	43.9 ± 13.3 (23–67)	43.9 ± 13.5 (19–75)	0.98
Female, male	26 (49%), 27(51%)	35 (44%), 45 (56%)	0.55
BMI (kg/m^2^)†	23.0 ± 3.6 (17.6–34.0)	26.3 ± 4.8 (18.8–45.7)	0.0002*
ODI	0.8 ± 2.2(0–10)	26.2 ± 15.2 (2–74)	< 0.0001*
VAS	1.1 ± 1.3 (0–6)	6.4 ± 2.4(1–10)	< 0.0001*

Sample size	*n* = 260 discs	*n* = 390 discs	

*Pfirrmann grade*			
I	64 (25%)	37 (10%)	< 0.0001*
II	114 (44%)	172 (44%)	
III	60 (23%)	82 (21%)	
IV	14 (5%)	72 (18%)	
V	8 (3%)	27 (7%)	
*Tiρ (ms)*			
NP region	76.4 ± 22.2 (40.8–145.0)	70.8 ± 22.8 (34.6–148.2)	0.009*
Whole disc	63.6 ± 15.3 (37.1–104.4)	60.6 ± 16.7 (33.2–138.1)	0.049*
*T2 (ms)*			
NP region	65.2 ± 19.4 (31.9–140.0)	60.4 ± 20.8 (30.6–152.4)	0.020*
Whole disc	54.2 ± 13.1 (30.6–102.7)	51.4 ± 14.8 (29.4–110.4)	0.058

Data are presented as mean ± SD (min-max) or number (percent of total). Group demographic differences were assessed using Student’s *t* tests (age, ODI, VAS, and BMI), a chi-square test (sex), a Cochran Armitage trend test (Pfirrmann grade), and mixed effects linear regression (T1ρ and T2)

*BMI* body mass index, *ODI* Oswestry Disability Index, *VAS* Visual Analog Scale, *NP* nucleus pulposus

* Indicates two-sided *p* < 0.05

† BMI data available for 39/53 (74%) of controls
