# Supplementary material for: ISSLS Prize in Bioengineering Science 2023: Age- and sex-related differences in lumbar intervertebral disc degeneration between patients with chronic low back pain and asymptomatic controls
Source: Eur Spine J. Author manuscript; Available in PMC 2023 May 24. (PMC10205694; doi:10.1007/s00586-023-07542-6)
Supplement: 1895942_Sup2 [file NIHMS1895942-supplement-1895942_Sup2.pdf]

Age- and sex-related differences in lumbar intervertebral disc degeneration between patients with chronic low back pain and asymptomatic controls

Supplemental material 2: parameter study using T2 relaxation-time mapping and comparisons with T1ρ

Methods

T2 relaxation-time maps were computed analogously to the methods used for T1ρ relaxation times, by fitting the signal decay of each voxel to the mono-exponential decay functions: $SI_i(TE) = S_0 e^{TE/T2}$, where TE is the echo time. As the T1ρ and T2 mapping sequences were acquired using a combined acquisition and have equivalent image matrices, the same segmentation masks and NP regions were used to compute NP-T2 values. The correlation between T1ρ and T2 relaxation times were assessed on a voxel-by-voxel basis and using the mean value in the NP.

To assess the specificity of our results to T1ρ MRI, the statistical tests involving NP-T1ρ (mixed- and fixed-effects linear regression) were repeated using NP-T2 in place of NP-T1ρ.

Results

There were strong positive correlations between T1ρ and T2 relaxation times both on a voxel-by-voxel basis (Figure S1a) and using mean values computed for the NP (Figure S1b). The correspondence between T1ρ and T2 was weaker at higher values (Figure S1c).

In the mixed effects regression model, NP-T2 was statistically significantly associated with the same main effects as NP-T1ρ (age, sex, spinal level, and group; p = .02–.0001; Table S1). However, the three-way interaction term age × level × group, which was statistically significantly associated with NP T1ρ (p = .02), was not significantly associated with NP-T2 (p =

.08). In the fixed effects regression models performed for each level separately, all statistically significant terms found using NP-T1ρ were also statistically significant using NP-T2.

Supplemental figures and tables

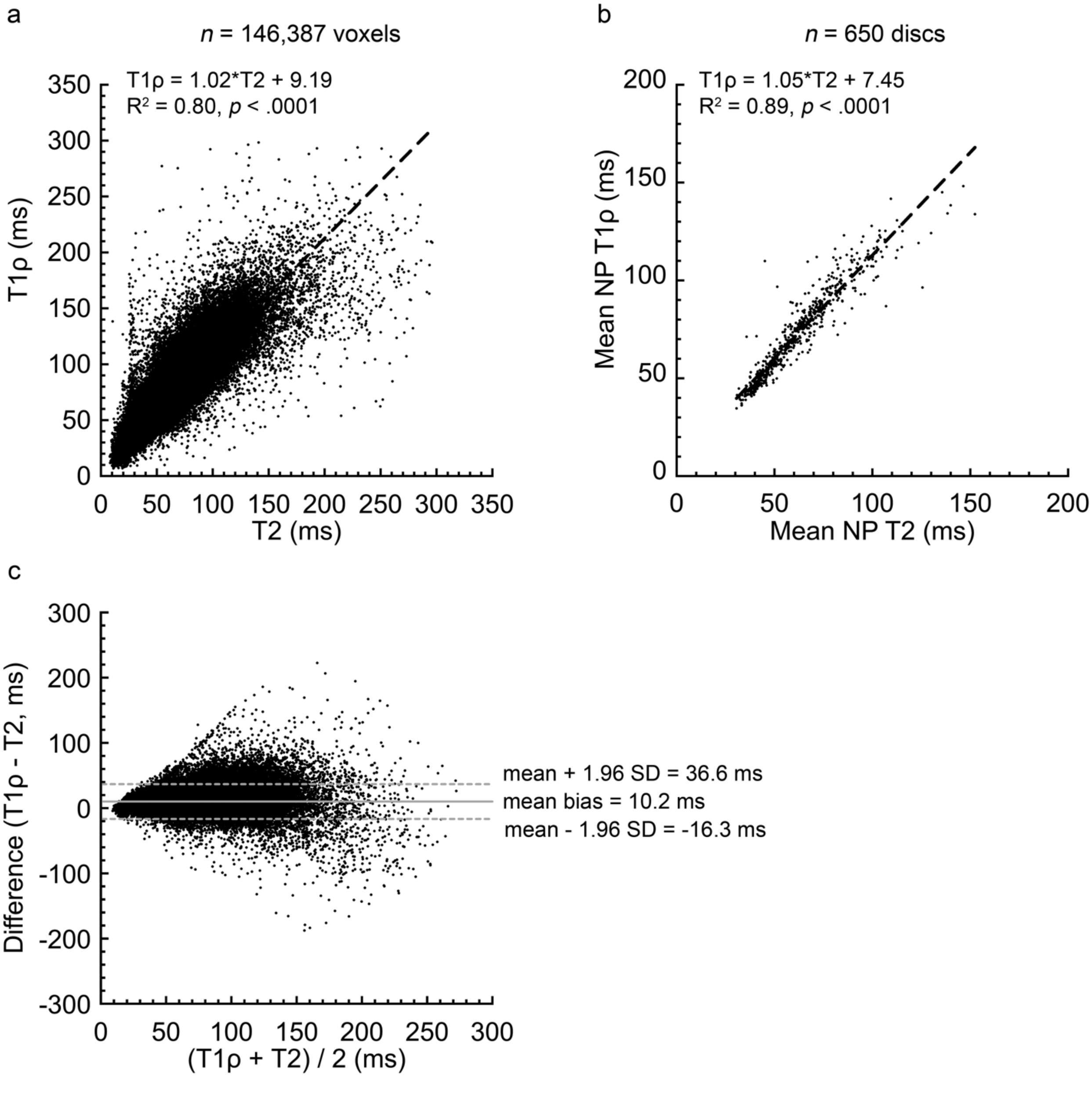

Fig. S1 Correlations between T1ρ and T2 on (a) a voxel-by-voxel basis and (b) using mean values computed in the NP. The Bland-Altman plot (c) demonstrates that correspondence was weaker at higher relaxation-time values

Table S1 Mixed-effects regression terms predicting NP-T1ρ/T2 (dependent variable). Bold typeface indicates the interaction term that was statistically significantly associated with NP-T1ρ but not NP-T2. * indicates two-sided $p < .05$

	p-value		
**Term**	**NP-T1ρ**	**NP-T2**	**Statistical conclusion**
Age	<.0001*	<.0001*	Not different
Level	<.0001*	<.0001*	Not different
Group	0.0085*	0.0204*	Not different
Sex	0.0001*	0.0006*	Not different
Sex × Group	0.1509	0.1033	Not different
Age × Group	0.3454	0.4115	Not different
Age × Sex	0.3738	0.3072	Not different
Age × Level	<.0001*	<.0001*	Not different
Sex × Level	0.0511	0.1099	Not different
Level × Group	0.1134	0.2522	Not different
Age × Sex × Level	0.5222	0.3906	Not different
Age × Sex × Group	0.8141	0.975	Not different
**Age × Level × Group**	**0.0172***	**0.08**	**Different**
Sex × Level × Group	0.0744	0.1476	Not different
Age × Sex × Level × Group	0.4476	0.3177	Not different
