# Supplementary material for: ISSLS Prize in Bioengineering Science 2023: Age- and sex-related differences in lumbar intervertebral disc degeneration between patients with chronic low back pain and asymptomatic controls
Source: Eur Spine J. Author manuscript; Available in PMC 2023 May 24. (PMC10205694; doi:10.1007/s00586-023-07542-6)
Supplement: 1895942_Sup1 [file NIHMS1895942-supplement-1895942_Sup1.pdf]

Age- and sex-related differences in lumbar intervertebral disc degeneration between patients with chronic low back pain and asymptomatic controls

Supplemental material 1: MRI acquisition parameters

Participants were imaged with 3.0 T MRI (Discovery MR 750 scanner, GE Healthcare) using an 8-channel phased-array spine coil. Sagittal acquisitions of the lumbar spine included clinical fast spin echo (FSE) images with T1 and T2 weighting, and a combined T1ρ and T2 relaxation-time mapping sequence with a segmented 3D spoiled gradient recalled (SPGR) acquisition (MAPPS)[1]. The sequence details are as follows:

1. Clinical fast spin echo (FSE) images using the following parameters for T1-, T2-weighting, respectively: TE = 15, 60 ms; TR = 511, 4788 ms; FOV = 26 cm; slice thickness = 3 mm; and in-plane resolution = 0.5 mm.
2. T1ρ magnetization preparation was performed using four spin-lock times (TSL = 0, 10, 40, 80 ms) and the T2 preparation was performed with four echo times (TE = 0, 8, 16, 64 ms). The acquisition parameters were: repetition time (TR) = 5.2 ms, field of view (FOV) = 20 cm, in-plane resolution = 0.8 mm, slice thickness = 8 mm; spin lock frequency = 300 Hz.

---

[1] Li X, Han ET, Busse RF, et al. In vivo T1ρ mapping in cartilage using 3D magnetization-prepared angle-modulated partitioned k-space spoiled gradient echo snapshots (3D MAPSS). *Magn Reson Med*. 2008;59(2):298–307.
